# Maximizing the therapeutic benefits of biopolymer-derived nanoparticles in wound healing

**DOI:** 10.22038/ijbms.2025.82225.17787

**Published:** 2025

**Authors:** Seyedeh Farnaz Darghiasi, Fatemeh Rajabi, Ashkan Farazin

**Affiliations:** 1 Department of Mechanical & Biomedical Engineering, Boise State University, Boise, ID, USA; 2 Biomolecular Sciences Graduate Programs, Boise State University, Boise, USA; 3 Department of Mechanical Engineering, Stevens Institute of Technology, 1 Castle Point Terrace, Hoboken, NJ 07030, USA

**Keywords:** Antibacterial properties, Biopolymer, Nanofibers, Nanoparticles, Wound healing

## Abstract

Nanoparticles have emerged as a cornerstone of nanomedicine, offering transformative potential to modern healthcare through their multifunctional capabilities. Their adaptability positions them as ideal candidates for wound management, either as advanced wound dressings or as efficient drug delivery systems. With intrinsic antibacterial properties and the ability to enhance tissue repair, nanoparticles have gained significant attention in promoting effective wound healing. Biopolymer-based nanoparticles, derived from naturally sourced and synthetic materials such as proteins, polysaccharides, and polymers, including collagen, chitosan, alginate, polycaprolactone, and polylactic acid, stand out due to their unique combination of biodegradability and biocompatibility. These attributes make them particularly suited for addressing the challenges of wound care. Moreover, nanofibers incorporated with biopolymer-based nanoparticles demonstrate superior antibacterial properties and wound healing effectiveness, comparable to the performance of silver nanoparticles. These advancements signify a transformative approach in wound healing therapies, facilitating targeted and personalized treatments that expedite tissue regeneration and enhance patient recovery. This review delves into biopolymer-based nanoparticles’ advancements, applications, and potential in revolutionizing wound healing.

## Introduction

Wound healing is a complex and dynamic process encompassing several overlapping stages: hemostasis, inflammation, proliferation, and tissue remodeling (1). Any disruption in these phases can cause delayed wound healing, the development of chronic wounds, or excessive scar formation, leading to considerable morbidity and an increased healthcare burden (2). Conventional wound management approaches often involve the use of dressings, topical agents, or surgical interventions, which may be limited by issues such as poor drug retention, inadequate tissue penetration, or systemic side effects (3). In recent years, nanotechnology has emerged as a transformative platform in wound care, overcoming these challenges and providing innovative therapeutic solutions (4). Biopolymer-based nanoparticles, with their distinctive properties and versatile functionalities, have gained significant attention for their potential to address the limitations of traditional wound healing approaches (5). One of the primary benefits of biopolymer-based nanoparticles is their ability to encapsulate and effectively deliver various therapeutic agents—including small molecules, peptides, proteins, and nucleic acids—while enhancing their stability and bioavailability (6). For example, growth factors like platelet-derived growth factor (PDGF), transforming growth factor-beta (TGF-β), and vascular endothelial growth factor (VEGF) are essential in promoting angiogenesis, collagen production, and epithelialization, all crucial processes in wound repair (7). Nevertheless, their short half-lives and rapid degradation *in vivo *highlight the need for advanced delivery systems that ensure sustained release and targeted, localized delivery (8). Biopolymer-based nanoparticles offer an attractive solution by providing a protective microenvironment for encapsulated growth factors, enabling controlled release kinetics and prolonged bioactivity at the wound site. Additionally, biopolymer-based nanoparticles can be designed with inherent antimicrobial properties, reducing the risk of wound infections, a frequent complication in delayed or impaired wound healing (5).

Polysaccharides such as chitosan and alginate exhibit intrinsic antimicrobial activity against a broad spectrum of pathogens, including bacteria, fungi, and viruses, making them suitable candidates for incorporation into nanoparticulate systems (9). By loading antimicrobial agents onto or within biopolymer-based nanoparticles, synergistic effects can be achieved, resulting in enhanced efficacy against drug-resistant microorganisms and biofilms commonly encountered in chronic wounds (5). Moreover, the adjustable physicochemical characteristics of biopolymer-based nanoparticles, including their size, shape, surface charge, and surface chemistry, allow for precise regulation of their interactions with biological systems, such as cells, proteins, and extracellular matrix components (10). This tunability facilitates the design of nanoparticles with optimized biodistribution, cellular uptake, and tissue retention profiles, thereby maximizing therapeutic outcomes while minimizing off-target effects (11). In conclusion, biopolymer-based nanoparticles represent a highly promising and versatile therapeutic platform for enhancing wound healing. Their multifunctional capabilities, such as targeted drug delivery, antimicrobial action, and modulation of the wound microenvironment, offer significant potential to address the unmet challenges in wound care, making them invaluable tools for clinicians and researchers alike (12). Despite substantial advancements in preclinical research, the clinical adoption of biopolymer-based nanoparticles for wound healing remains challenging due to scalability, regulatory hurdles, and cost-effectiveness (5). Overcoming these challenges will necessitate collaborative efforts among academia, industry, and regulatory bodies to drive the development and commercialization of nanoparticle-based wound therapies to enhance patient outcomes and overall quality of life (13, 14). 

Despite significant advancements, translating biopolymer-based nanoparticles into clinical applications for wound healing remains challenging due to scalability, regulatory approval, and cost-effectiveness. This review seeks to address these gaps by synthesizing the latest developments in the field, focusing on the multifunctional roles of biopolymer-based nanoparticles in drug delivery, antimicrobial activity, and modulation of the wound microenvironment. By emphasizing recent innovations and interdisciplinary collaborations, this work aims to understand their potential to revolutionize wound care comprehensively. Furthermore, it critically analyzes challenges and opportunities, providing actionable insights to accelerate clinical translation and improve patient outcomes. 

## Biopolymers and nanoparticles emerge as a blessing for the field of wound healing

Biopolymer-based nanoparticles facilitate wound healing by leveraging their biocompatible and biodegradable nature to deliver bioactive molecules effectively to the wound site. These nanoparticles can encapsulate growth factors such as VEGF and TGF-β, protecting them from rapid degradation and enabling sustained release. This controlled release enhances angiogenesis, collagen synthesis, and epithelialization, which is critical for efficient tissue regeneration. Furthermore, the surface chemistry of biopolymer-based nanoparticles can be engineered to interact with cell receptors, modulating cellular behaviors such as migration and proliferation. Recent studies have demonstrated that chitosan nanoparticles loaded with antimicrobial peptides accelerate wound closure and significantly reduce infection rates by disrupting bacterial membranes.

Collagen serves as a natural scaffold for cell adhesion, migration, and proliferation, promoting tissue regeneration and angiogenesis; chitosan exhibits inherent antimicrobial and hemostatic properties, stimulating fibroblast activity and enhancing wound closure; alginate, a polysaccharide from seaweed, creates a moist wound environment, absorbs exudates, and supports cell proliferation; polycaprolactone (PCL), a biodegradable polyester, provides mechanical strength and long-term structural support for tissue regeneration; and polylactic acid (PLA), a synthetic polymer, degrades into lactic acid, aiding tissue remodeling while enabling sustained and localized drug delivery all of which, when combined with nanoparticles, improve wound healing outcomes through enhanced bioactivity, antimicrobial action, and controlled therapeutic release.

**Figure 1:**
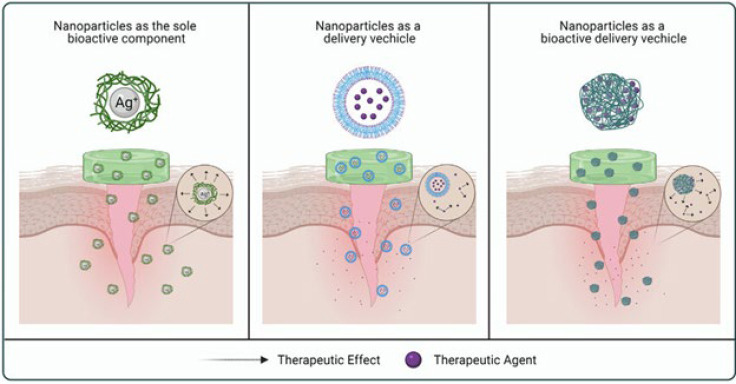
Nanoparticles can act as sole bioactive components providing therapeutic effects (e.g., silver nanoparticles for antimicrobial action), as delivery vehicles enabling targeted and controlled release of therapeutic agents at the wound site, or as bioactive delivery vehicles combining their inherent bioactivity with encapsulated agents for synergistic wound healing effects [30]

Biopolymers and nanoparticles stand at the forefront of innovation in wound healing, presenting a symbiotic alliance with immense promise for revolutionizing therapeutic approaches (15). Wound management, spanning from acute injuries to chronic ulcers, poses a significant clinical challenge worldwide (16). Traditional treatment modalities often fall short in addressing the complex pathophysiology of wounds, necessitating the exploration of novel strategies (17). Biopolymers, sourced from natural materials like proteins, polysaccharides, and nucleic acids, are highly biocompatible and biodegradable, making them ideal for biomedical uses. Meanwhile, nanoparticles, with their customizable physicochemical properties and multifunctionality, serve as an excellent platform for applications such as targeted and controlled drug delivery (18). The synergy between biopolymers and nanoparticles capitalizes on their respective strengths, offering a multifaceted approach to wound healing (19). Biopolymers are the backbone for nanoparticle synthesis, providing a biologically compatible matrix for drug encapsulation and delivery (20). By entrapping bioactive molecules within nanoparticles, biopolymers shield them from degradation, extend their retention time at the wound site, and facilitate controlled release kinetics, optimizing therapeutic efficacy (5). Additionally, biopolymer-based nanoparticles can be functionalized with targeting ligands or stimuli-responsive elements, enabling site-specific delivery and controlled, on-demand release of therapeutics, increasing their precision and effectiveness (21). Beyond drug delivery, biopolymer-based nanoparticles exhibit inherent properties that actively support and enhance wound healing (22). Polysaccharides like chitosan and alginate possess natural antimicrobial properties, making them particularly effective in preventing wound infections, which are a frequent complication in impaired wound healing (23). Furthermore, biopolymer-based nanoparticles can influence the wound microenvironment by interacting with cells and extracellular matrix components, thereby enhancing cellular proliferation, migration, and differentiation—critical processes for effective tissue regeneration and repair (24). By leveraging these intrinsic properties, biopolymer-based nanoparticles provide a comprehensive approach to wound healing, effectively targeting multiple stages of the wound healing process simultaneously (2). The versatility of biopolymer-based nanoparticles extends beyond traditional wound healing modalities, encompassing emerging therapeutic strategies such as gene therapy and regenerative medicine (10). By encapsulating nucleic acids or growth factors within nanoparticles, biopolymers facilitate their delivery to target cells, enabling the modulation of cellular processes involved in wound healing (25). Additionally, biopolymer-based nanoparticles can act as scaffolds for tissue engineering, offering a three-dimensional matrix that supports cell attachment, proliferation, and differentiation, thereby promoting tissue regeneration and functional recovery (26). Despite the significant promise of biopolymer-based nanoparticles in wound healing, several hurdles must be overcome. Key challenges include scalability, reproducibility, safety, and regulatory approval, all of which are essential for the successful clinical translation of nanoparticle-based therapies (27). Moreover, the intricate interactions between nanoparticles and biological systems require a comprehensive understanding of their pharmacokinetics, biodistribution, and toxicity profiles to guarantee their safety and effectiveness in clinical applications (28). In conclusion, the synergy between biopolymers and nanoparticles represents a paradigm shift in wound healing therapeutics, offering a versatile and multifaceted approach to address unmet needs in wound care (27). By capitalizing on the distinctive properties of biopolymers and nanoparticles, researchers and clinicians can devise innovative approaches to accelerate tissue regeneration, prevent wound infections, and enhance patient outcomes (29). However, realizing the full potential of biopolymer-based nanoparticles in wound healing requires collaborative efforts from multidisciplinary teams spanning academia, industry, and regulatory agencies to overcome existing challenges and translate benchtop innovations into clinical reality, as shown in Figure 1.

## Function of polymeric nanoparticles in the process of wound healing

**Figure 2:**
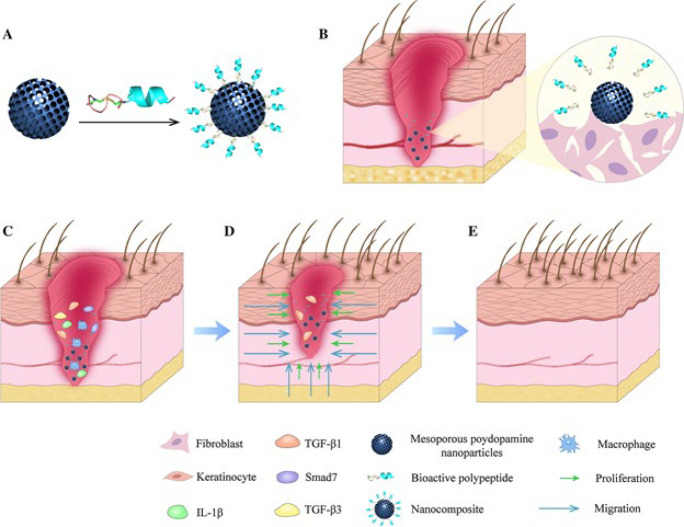
Schematic diagram of delivery system to improve the healing

Polymeric nanoparticles are instrumental in wound healing, providing a multifaceted strategy to tackle the intricate challenges of tissue repair and regeneration (31). These nanoparticles, derived from biocompatible and biodegradable polymers, exhibit unique properties that make them promising candidates for therapeutic intervention in wound management (32). A key function of polymeric nanoparticles in wound healing is their ability to act as highly effective carriers for delivering bioactive molecules directly to the injury site (33). These molecules may include growth factors, antimicrobial agents, anti-inflammatory drugs, and nucleic acids, among others (34). Encapsulating these therapeutic agents within polymeric nanoparticles enhances their stability, and controlled release kinetics are achieved, allowing for sustained and localized delivery to the wound microenvironment (35). This targeted delivery approach promotes tissue regeneration, reduces inflammation, and prevents infection, accelerating the healing process (12). Moreover, polymeric nanoparticles can modulate the wound microenvironment by interacting with cells and extracellular matrix components. Surface modifications of nanoparticles with specific ligands enable them to target and bind to cell receptors, facilitating cellular uptake and intracellular delivery of therapeutic payloads (25). This interaction stimulates cellular proliferation, migration, and differentiation, which is essential for effective tissue repair and regeneration (36). Additionally, polymeric nanoparticles can mimic the extracellular matrix, providing a scaffold for cell attachment and growth, further enhancing tissue regeneration at the wound site (37). Furthermore, polymeric nanoparticles possess inherent antimicrobial properties, particularly when derived from polymers such as chitosan, alginate, or poly(lactic-co-glycolic acid) (PLGA) (33). These nanoparticles can directly inhibit the growth of bacteria, fungi, and other pathogens commonly associated with wound infections. By incorporating antimicrobial agents or peptides into polymeric nanoparticles, synergistic effects can be achieved, resulting in enhanced efficacy against drug-resistant microorganisms and biofilms, thus preventing and treating wound infections effectively (38). Beyond their therapeutic functions, polymeric nanoparticles provide benefits such as biocompatibility, biodegradability, and customizable physicochemical properties, making them highly adaptable for a wide range of wound healing applications (39). Their small size allows for deep tissue penetration and cellular uptake. At the same time, their surface can be functionalized by targeting ligands, imaging agents, or stimuli-responsive moieties to enhance their specificity and functionality (40). Furthermore, polymeric nanoparticles can be formulated into different delivery systems, including hydrogels, films, and dressings, to provide sustained release and prolonged therapeutic effects (39). In summary, polymeric nanoparticles play a pivotal role in wound healing by facilitating targeted drug delivery, modulating the wound microenvironment, and preventing microbial infections (12). Their versatile properties and customizable functionalities make them valuable tools for accelerating the healing process and improving outcomes in both acute and chronic wounds (41). However, further research is needed to optimize their formulation, enhance their efficacy, and ensure their safety for clinical translation, ultimately advancing the field of wound care, as shown in Figure 2.

## Improved drug transportation

Enhanced drug delivery using nanoparticles holds immense potential for improving the efficacy of existing drugs and enabling the delivery of therapeutic molecules that were previously deemed impractical due to their physicochemical properties or systemic toxicity (43). For instance, nanoparticles can encapsulate hydrophobic drugs within their lipid bilayers or hydrophilic drugs within their aqueous cores, overcoming solubility issues and enhancing bioavailability (44). Additionally, nanoparticles can shield drugs from enzymatic degradation or harsh physiological conditions, extending their circulation time and enhancing their therapeutic efficacy (43). In treating infectious diseases, nanoparticles provide distinct advantages by enabling targeted drug delivery directly to the sites of infection (45). Functionalization of nanoparticles with ligands that recognize specific microbial surface antigens or host cell receptors allows for selective targeting of infected tissues, minimizing off-target effects and reducing the risk of antimicrobial resistance (46). Furthermore, nanoparticles can penetrate bacterial biofilms, which are notoriously resistant to conventional antibiotics, effectively eradicating persistent infections (47). In inflammatory conditions like rheumatoid arthritis or inflammatory bowel disease, nanoparticles can be designed to deliver anti-inflammatory drugs directly to inflamed tissues, reducing systemic side effects associated with prolonged drug exposure (48). Moreover, nanoparticles can modulate immune responses by selectively targeting immune cells or delivering immunomodulatory agents, offering potential therapeutic benefits in autoimmune diseases and transplant rejection (49). Beyond small molecule drugs, nanoparticles hold promise for delivering biologics such as nucleic acids, peptides, and proteins, which often face challenges related to stability, delivery, and immunogenicity (50). Nanoparticles can protect these fragile molecules from degradation, facilitate their cellular uptake, and enable their intracellular delivery to exert therapeutic effects (43). This is particularly relevant in gene therapy, where nanoparticles can deliver nucleic acids such as DNA or RNA to target cells, offering potential treatments for genetic disorders, cancer, and infectious diseases (51). Moreover, the advancement of personalized medicine is deeply dependent on improved drug delivery technologies (52). Nanoparticles can be customized to match individual patient characteristics, including genetic profile, disease progression, and treatment response, allowing for precise dosing and highly targeted therapeutic interventions (53). By integrating diagnostics with therapeutics, nanoparticles enable theranostic applications, combining drug delivery and imaging into a single platform for real-time monitoring of disease progression and treatment efficacy. In summary, nanoparticle-based drug delivery represents a groundbreaking advancement in drug development, offering wide-ranging applications across diverse medical conditions (43). 

## Antimicrobial properties of nanoparticles for wound healing

**Figure 3:**
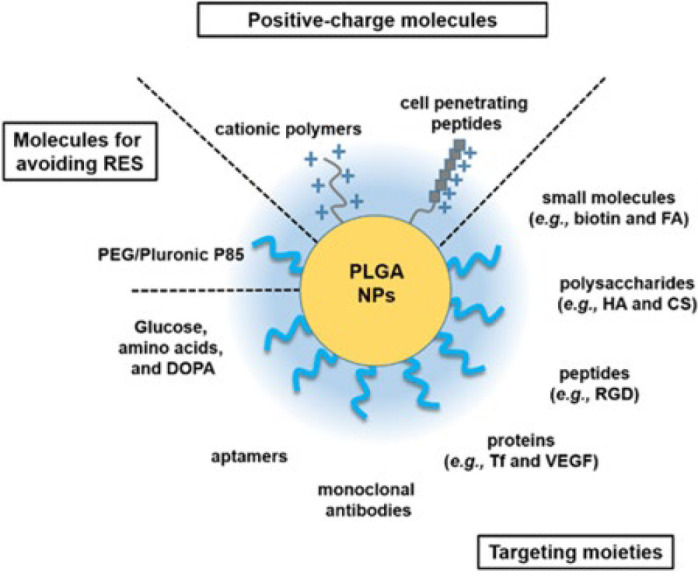
Illustrates the multifunctionality of PLGA nanoparticles, showcasing their surface modifications with positive-charge molecules (e.g., cationic polymers, cell-penetrating peptides) for enhanced cellular uptake, molecules like PEG and DOPA to evade immune clearance, and targeting moieties (e.g., biotin, RGD peptides, HA, VEGF, and monoclonal antibodies) for precise and efficient delivery to specific cells or tissues [62]

The antimicrobial properties of nanoparticles have emerged as a promising strategy for enhancing wound healing outcomes, particularly in the context of combating infections (54). Nanoparticles exhibit distinctive physicochemical properties that enable them to act as potent antimicrobial agents, effective against a wide range of pathogens, including bacteria, fungi, and viruses (55). One of the key advantages of nanoparticles in wound healing is their high surface area-to-volume ratio, which enables efficient interaction with microbial cells and disrupts their membranes, leading to cell lysis and death (13). Additionally, nanoparticles can penetrate bacterial biofilms, which are notoriously resistant to conventional antimicrobial agents, thereby overcoming a significant barrier to effective wound treatment (47). Various types of nanoparticles, including metallic nanoparticles (e.g., silver, copper), metal oxide nanoparticles (e.g., zinc oxide, titanium dioxide), and polymer-based nanoparticles (e.g., chitosan, poly(lactic-co-glycolic acid) (PLGA)) as shown Figure 3, have demonstrated potent antimicrobial activity in preclinical studies (56). These nanoparticles can be incorporated into wound dressings, creams, or hydrogels to provide sustained release of antimicrobial agents at the wound site, thereby preventing or treating infections while promoting tissue regeneration (9). Furthermore, nanoparticles can be functionalized with antimicrobial peptides, enzymes, or antibodies to enhance their specificity and efficacy against target pathogens (57). By conjugating targeting ligands to nanoparticles, selective antimicrobial activity can be achieved, minimizing off-target effects on beneficial commensal microorganisms and reducing the risk of antimicrobial resistance (58). The use of antimicrobial nanoparticles in wound healing offers several advantages over conventional antimicrobial agents, including enhanced stability, prolonged activity, and reduced toxicity (59). Additionally, nanoparticles can work synergistically with other wound healing strategies, such as growth factors or anti-inflammatory drugs, to enhance and optimize wound healing outcomes (60). However, the clinical translation of antimicrobial nanoparticles for wound healing faces several challenges, including issues of scalability, ensuring biocompatibility, and obtaining regulatory approval (61). Further research is needed to optimize nanoparticle formulations, evaluate their safety profiles, and conduct rigorous clinical trials to demonstrate their efficacy in real-world settings. In conclusion, the antimicrobial properties of nanoparticles offer significant potential to enhance wound healing by effectively preventing or treating infections while supporting tissue regeneration (54). Continued research and innovation in nanoparticle-based antimicrobial therapies are essential for addressing the unmet needs in wound care and advancing patient care.

## Acceleration of the healing process with biopolymer and nanoparticles

The synergistic combination of biopolymers and nanoparticles to accelerate the healing process marks a significant breakthrough in wound care (29). Biopolymers, sourced from natural materials like proteins, polysaccharides, and nucleic acids, inherently offer biocompatibility and biodegradability, making them excellent candidates for supporting tissue repair and regeneration (63). Combined with nanoparticles, which offer unique physicochemical properties and versatile functionalities, these biopolymers can enhance therapeutic outcomes in various stages of wound healing. One of the primary mechanisms by which biopolymer-based nanoparticles accelerate the healing process is through the controlled delivery of bioactive molecules to the wound site (6). Growth factors, cytokines, and other signaling molecules are essential in coordinating the intricate series of events in tissue repair, such as cell proliferation, migration, and differentiation (64). Encapsulating bioactive molecules within nanoparticles or conjugating them to biopolymer-based carriers improves their stability and bioavailability, allowing for sustained release and targeted delivery to the wound microenvironment (65). This targeted delivery strategy enhances angiogenesis, collagen production, and epithelialization, resulting in accelerated wound closure and improved tissue remodeling (66). Moreover, biopolymer-based nanoparticles can modulate the wound microenvironment to create a favorable milieu for healing (24). These nanoparticles engage with cells, extracellular matrix components, and immune mediators to regulate inflammatory responses and support tissue regeneration. For instance, nanoparticles made from chitosan or hyaluronic acid can influence macrophages and fibroblasts, encouraging a transition from a pro-inflammatory to a pro-regenerative phenotype (25). Additionally, nanoparticles can replicate the structural and biochemical signals of the native extracellular matrix, serving as a scaffold to support cell adhesion, migration, and proliferation, thereby further promoting tissue repair processes (67). Furthermore, the antimicrobial properties of biopolymer-based nanoparticles contribute to preventing and treating wound infections, which can significantly delay the healing process (6). Polysaccharide-based nanoparticles, such as chitosan or alginate, possess intrinsic antimicrobial activity against a wide range of pathogens, including bacteria, fungi, and viruses (68). By incorporating antimicrobial agents or peptides into these nanoparticles, synergistic effects can be achieved, enhancing efficacy against drug-resistant microorganisms and biofilms commonly encountered in chronic wounds (38). Beyond their therapeutic roles, biopolymer-based nanoparticles provide benefits such as biocompatibility, customizable properties, and ease of functionalization, making them highly versatile platforms for personalized and precision wound care (27). Interdisciplinary collaborations among materials scientists, bioengineers, clinicians, and regulatory agencies can accelerate the clinical translation of biopolymer-based nanoparticles for wound healing, ultimately enhancing patient outcomes and quality of life (10).

## Recent research of specific biopolymers to improve wound healing

Recent studies have delved into the realm of wound healing using selective biopolymers, marking a significant advancement in this field (69). Biopolymers, sourced from natural materials like proteins, polysaccharides, and nucleic acids, provide a distinct approach to enhancing tissue repair and regeneration due to their natural biocompatibility and bioactivity (70). These studies have focused on harnessing the therapeutic potential of specific biopolymers to address various aspects of the wound-healing process, including inflammation, angiogenesis, and tissue remodeling (71). A notable area of interest in recent research focuses on chitosan, a polysaccharide derived from chitin commonly found in the exoskeletons of crustaceans (69). Chitosan exhibits antimicrobial properties and promotes hemostasis, making it particularly suitable for managing wounds prone to infection (72). Recent research has explored the incorporation of chitosan into wound dressings, hydrogels, and scaffolds to enhance wound closure, reduce inflammation, and prevent microbial colonization (73). Furthermore, chitosan nanoparticles have been developed to directly deliver bioactive molecules such as growth factors and antimicrobial agents to the wound site, improving therapeutic efficacy while minimizing systemic side effects (74). Another promising biopolymer for wound healing applications is hyaluronic acid (HA), a glycosaminoglycan abundant in the extracellular matrix of connective tissues (75). HA plays a crucial role in regulating inflammation, angiogenesis, and tissue regeneration, making it an attractive candidate for promoting wound healing. Recent studies have investigated using HA-based hydrogels, films, and nanoparticles to enhance wound repair by delivering growth factors, cytokines, and stem cells. HA-based dressings have shown promise in accelerating wound closure, reducing scar formation, and improving overall tissue regeneration in acute and chronic wounds (76). In addition to chitosan and HA, other biopolymers such as collagen, gelatin, and alginate have also been explored for their potential in wound healing (23). Collagen, the primary structural protein in the extracellular matrix, serves as a natural scaffold for cell adhesion, migration, and proliferation, making it an excellent substrate for tissue engineering and regenerative medicine applications (77). Gelatin, derived from collagen, offers similar properties and has been used to develop biocompatible wound dressings and scaffolds for promoting wound healing (77). Alginate, a polysaccharide derived from brown seaweed, forms hydrogels capable of absorbing wound exudate, maintaining a moist environment, and supporting cell proliferation, making it an effective material for managing chronic wounds like diabetic ulcers and pressure sores (78). Recent studies on wound healing with selective biopolymers highlight the potential of these natural materials to enhance therapeutic outcomes and improve patient care (79). By harnessing the unique properties of biopolymers and advancing their use through innovative biomaterials and delivery systems, researchers strive to address unmet needs in wound management and expedite the translation of these technologies from research to clinical practice.

## Nanoparticle-enhanced nanofibers and hydrogels: advancements in wound healing

Nanoparticle-integrated nanofibers and hydrogels have emerged as innovative and effective strategies for improving wound healing outcomes. These advanced biomaterials combine the unique properties of nanoparticles with the structural support of nanofibers or the moisture-retaining capability of hydrogels to create innovative wound dressings with multifunctional properties (80). Nanofibers are fibrous structures with nanometer-scale diameters that closely replicate the extracellular matrix (ECM) architecture found in native tissues (81). Nanofibers feature high surface area-to-volume ratios and porosity, making them an excellent scaffold for cell attachment, migration, and proliferation. When nanoparticles are incorporated into these nanofibers, they enable the targeted delivery of therapeutic agents directly to the wound site, enhancing tissue regeneration and expediting the healing process (82). One approach involves electrospinning, which produces nanofibers from polymer solutions or melts (83). By incorporating nanoparticles into the polymer solution before electrospinning, nanoparticles can be uniformly dispersed within the resulting nanofibers (84). These nanoparticles can be loaded with bioactive molecules, including growth factors, antimicrobial agents, or anti-inflammatory drugs, allowing for sustained release and targeted delivery to the wound microenvironment (60). Hydrogels, conversely, are three-dimensional networks of hydrophilic polymers capable of absorbing large amounts of water (83). They create a moist environment at the wound site, which is conducive to cell migration, proliferation, and tissue regeneration. Incorporating nanoparticles into hydrogels can enhance their mechanical properties, stability, and bioactivity, leading to improved wound-healing outcomes (85). Nanoparticle-mediated nanofibers and hydrogels offer several advantages for wound healing applications (84). Firstly, they provide sustained release of therapeutic agents, ensuring prolonged exposure to bioactive molecules at the wound site. This can promote angiogenesis, collagen deposition, and epithelialization, leading to faster wound closure and reduced scar formation. Secondly, these biomaterials can be engineered to possess antimicrobial properties, preventing infections and promoting a sterile wound environment conducive to healing (86). Additionally, the biocompatibility and biodegradability of these materials minimize the risk of adverse reactions and facilitate wound dressing removal without causing trauma to the healing tissue (87). Additionally, nanoparticle-integrated nanofibers and hydrogels can be customized to address specific wound types and individual patient needs. By modifying their composition, structure, and functionalization, researchers can fine-tune these biomaterials to optimize their performance for various applications, including chronic wound care, burn treatment, and tissue engineering (88). Moreover, these innovative wound dressings can be integrated with other therapeutic approaches, such as cellular therapies or physical stimuli, to improve wound healing outcomes synergistically. In conclusion, nanoparticle-enhanced nanofibers and hydrogels offer promising platforms for advanced wound dressings with superior therapeutic potential. With ongoing research and development, these biomaterials can transform wound care practices and significantly enhance patient outcomes in the future (89).

## Current limitations and challenges of biopolymers and nanoparticles for wound healing

Despite the potential benefits, the practical application of biopolymers and nanoparticles in wound healing encounters significant barriers. Biocompatibility concerns warrant a thorough investigation, including potential immune reactions or allergic responses (90). Additionally, achieving precise control over the release kinetics of therapeutic agents from nanoparticles remains a significant challenge, necessitating innovative strategies to fine-tune delivery profiles. Another major hurdle is manufacturing scalability, as many nanoparticle fabrication techniques are currently limited to laboratory-scale production (91). Scaling up production without compromising nanoparticle integrity or efficacy is essential for clinical viability (44). Regulatory approval processes are also intricate and time-consuming, demanding rigorous safety and efficacy assessments to ensure patient well-being. Cost considerations pose yet another challenge, with the expense of biopolymer-based wound healing therapies and nanoparticle formulations potentially limiting their accessibility (92). Developing cost-effective production methods while maintaining product quality is imperative for widespread adoption. Additionally, ensuring the long-term stability of these formulations is crucial to uphold efficacy over extended periods, necessitating ongoing research into degradation mechanisms and storage conditions (93). Furthermore, unraveling the complex interactions between biopolymers, nanoparticles, and the wound microenvironment remains a critical area of investigation (94). Tailoring therapies to specific wound types and patient needs requires a deeper understanding of these interactions, guiding the development of personalized treatment approaches (95). Addressing these challenges will demand collaborative efforts from researchers, clinicians, industry partners, and regulatory agencies. Overcoming these challenges will pave the way for biopolymer-based wound healing therapies and nanoparticle formulations to revolutionize wound care, delivering better outcomes and significantly enhancing the quality of life for patients globally (92).

## Upcoming developments in wound healing therapies utilizing polymeric nanoparticles

In wound healing, emerging trends in polymeric nanoparticle-based therapies show great potential to transform treatment strategies and revolutionize patient care. These advancements are anticipated to address current challenges and enhance patient outcomes through several key avenues. Personalized medicine is expected to play a pivotal role, with nanotechnology enabling tailored treatments based on individual patient characteristics(96). Additionally, targeted drug delivery systems will become increasingly sophisticated, allowing for precise localization of therapeutic agents to the wound site while minimizing systemic side effects (97). Combination therapies, integrating multiple therapeutic modalities within nanoparticle carriers, will offer synergistic benefits for tissue regeneration and wound closure (98). Biomimetic materials inspired by the native extracellular matrix will enhance biocompatibility and functionality, promoting more effective wound healing (99). Additionally, incorporating advanced imaging and monitoring technologies will allow real-time assessment of treatment effectiveness and support the personalization of therapeutic strategies. Collectively, these emerging trends in polymeric nanoparticle-based wound healing therapies have the potential to significantly enhance patient care and outcomes (100).

## Smart nanoparticles for wound healing

Smart nanoparticles represent a state-of-the-art advancement in wound healing, providing customized solutions to effectively address the dynamic and intricate wound microenvironment (13). These nanoparticles are designed with advanced functionalities that enable them to respond intelligently to specific cues or stimuli present in the wound environment, thereby enhancing therapeutic outcomes (101). One of the key features of smart nanoparticles is their ability to target and deliver therapeutic agents with precision to the wound site. By functionalizing the nanoparticle surface by targeting ligands or antibodies, smart nanoparticles can selectively bind to cell receptors, or biomarkers overexpressed in injured tissues, ensuring localized delivery of therapeutic payloads (102). This targeted strategy reduces systemic side effects while maximizing therapeutic efficacy. Additionally, smart nanoparticles can respond to specific stimuli in the wound microenvironment, such as pH, temperature, or enzyme activity, enabling precise and controlled drug release (12). Stimuli-responsive nanoparticles are designed to undergo structural transformations or activate release mechanisms in response to specific triggers, facilitating on-demand drug delivery at the precise site and time (103). For instance, pH-responsive nanoparticles can release drugs when exposed to the acidic environment of inflamed or infected tissues. In contrast, temperature-sensitive nanoparticles are triggered to release drugs in response to elevated body temperatures associated with inflammation (104). Beyond targeted drug delivery and controlled release, smart nanoparticles can be equipped with diagnostic or imaging capabilities, enabling real-time monitoring of wound healing progress (105). For instance, fluorescent nanoparticles can be used to track the distribution and accumulation of nanoparticles within the wound site using fluorescence imaging techniques (106). Magnetic nanoparticles can be tracked using magnetic resonance imaging (MRI), providing a non-invasive method to monitor their biodistribution and drug release kinetics in real time (107). Additionally, smart nanoparticles can be designed with intrinsic therapeutic properties, such as antimicrobial activity or immunomodulatory effects, to support wound healing. These nanoparticles can achieve synergistic effects by encapsulating agents like antimicrobial compounds, growth factors, or anti-inflammatory drugs, promoting enhanced tissue regeneration and faster wound closure (13). Smart nanoparticles have tremendous potential for revolutionizing wound healing therapies by offering targeted, controlled, and personalized interventions (4). Continued research and innovation in nanoparticle design, fabrication, and functionalization will further advance the field, paving the way for more effective and efficient wound care strategies in the future.

Image from Gowda BHJ, Mohanto S, Singh A, Bhunia A, Abdelgawad MA, Ghosh S, *et al*. (2023), Mater Today Chem, licensed under CC BY 4.0

A Successful preparation of nanocomposites. B MPDA released RL-QN15 on the surface of the wound. C RL-QN15 induced cytokine secretion from macrophages. D Migration and proliferation of keratinocytes and fibroblasts induced by RL-QN15. E Wounds treated with nanocomposites of RL-QN15 healed completely [42]

Image from Liu R, Luo C, Pang Z, Zhang J, Ruan S, Wu M, *et al*. (2022), Chin Chem Lett, licensed under CC BY 4.0

Image from Kim KT, Lee JY, Kim DD, Yoon IS, Cho HJ. (2019), Pharmaceutics, licensed under CC BY 4.0

## Conclusion

Nanoparticles hold immense potential to transform the healthcare sector, particularly in wound healing, due to their versatile properties and practical applications. Their biodegradable and biocompatible nature makes them invaluable in modern medicine, with polymeric nanoparticles (PNPs) such as collagen, chitosan, and polylactic acid emerging as versatile tools. Utilized as wound dressings or drug delivery carriers, these nanoparticles possess antimicrobial properties that create an environment conducive to faster wound healing. Furthermore, their large surface area and capacity for targeted therapeutic delivery ensure precise and efficient treatment at the wound site. This evolution in wound care represents a shift toward patient-centered, personalized solutions aimed at promoting efficient tissue regeneration and expedited wound closure. As advanced diagnostic technologies and innovative materials are integrated into ongoing research, the future of wound care holds great promise for more effective and tailored treatments, heralding a new era of innovation in healthcare.

## Declaration

We have not used any AI tools or technologies to prepare this manuscript.
